# The Effects of the COVID-19 Pandemic on Healthcare Providers’ Mental Health: Experiences at Kenyatta National Hospital, Kenya

**DOI:** 10.3390/bs14050351

**Published:** 2024-04-23

**Authors:** Vallery Ogello, Nicholas Thuo, Phelix Okello, Njeri Wairimu, Paul Mwangi, Gakuo Maina, Harrison Mwenda, Paul Mutua, John Kinuthia, Linnet Ongeri, Nelly Mugo, Kenneth Ngure

**Affiliations:** 1 Partners in Health Research and Development, Centre for Clinical Research, Kenya Medical Research Institute, Nairobi 19865-00202, Kenya; 2 Kenyatta National Hospital, Nairobi 20723-00202, Kenya; 3 Kenya Medical Research Institute, Nairobi 54840-00200, Kenya; 4 Department of Global Health, University of Washington, Seattle, WA 98105, USA; 5 School of Public Health, Jomo Kenyatta University of Agriculture and Technology, Nairobi 62000-00200, Kenya

**Keywords:** mental health, COVID-19, healthcare providers, Kenyatta National Hospital

## Abstract

Background: In 2020, healthcare providers were expected to provide care to individuals with coronavirus disease 2019 (COVID-19), putting them at risk of acquiring COVID-19. The possibility of acquiring poorly understood infectious diseases while providing care may have an impact on the mental health of providers. We conducted a study to explore the effects of COVID-19 on the mental health of healthcare providers. Methods: Between April and August 2021, we conducted in-depth interviews with 60 healthcare providers in the infectious disease unit (IDU) and other units of the hospital (non-IDU). The healthcare providers completed an online self-administered survey form with demographic data (age, sex, average income, and known contact with a COVID-19 patient). We used semi-structured interview guides to understand the healthcare providers’ lived experiences of stress, anxiety, depression, and their associated factors. We transcribed the interviews verbatim and coded and analyzed the transcripts to derive thematic concepts related to mental health experiences. Results: The healthcare providers had a median age of 37 years [IQR 20.0–58.0], and 56.7% were female, 30.0% nurses, 18.3% medical doctors, and 11.7% laboratory technologists. The healthcare providers reported increased stress during the pandemic, attributed to the high demand for patient care, changes in social life, and fear of COVID-19 infection. They also reported experiences of anxiety and depression as a result of limited knowledge at the beginning of the pandemic and the perception that “COVID-19 resulted in death”. Testing positive for COVID-19, high exposure to COVID-19 risks, and the death of patients and colleagues reportedly affected the healthcare providers’ mental health. Additionally, the healthcare providers reported mental health support through debriefing meetings, peer-to-peer support, and psychological counseling, with privacy and confidentiality concerns. Conclusions: Healthcare providers faced mental health issues such as stress and anxiety while taking care of COVID-19 patients. An effective mental health response requires institutional practices that address context-specific challenges such as privacy and confidentiality.

## 1. Introduction

In 2020, 17 million people were reported to have acquired coronavirus disease 2019 (COVID-19), caused by severe acute respiratory coronavirus 2 (SARS-CoV-2), which resulted in over 600,000 deaths globally [1]. In Africa, the COVID-19 pandemic was confirmed to have spread into Egypt in February 2020, and in sub-Saharan Africa, Nigeria was the first infected country in the same month [2]. Kenya reported its first case in March 2020 and by May had reported over 800 cases and 50 deaths [3]. By 2020, initial estimates reported that frontline healthcare workers could account for 10–20% of all diagnoses [4]. Globally, in 2020, a total of 152,888 infections and 1413 deaths were reported among healthcare workers [5].

Health providers have been shown to have experienced mental health issues during the COVID-19 pandemic due to self-isolation, such as self-harm and depression. For instance, a study conducted in China found that healthcare workers working with individuals affected by, or at risk of, COVID-19 were at risk of adverse mental health consequences [6]. Subsequently, a web-based survey that included 31 countries across the globe and a review among low- and middle-income countries showed high levels of anxiety and depression at the onset of the pandemic among healthcare providers (HCPs) [7,8]. Regarding these increased anxiety levels, the World Health Organization (WHO) issued thirty-one-point considerations for mitigating anxiety, depression, and stigma, with a focus on service providers and their managers, people with pre-existing conditions, women, and children [9,10]. Further, HCPs in Africa also exhibited low job satisfaction and high levels of burnout and stress during the pandemic [11]. Additionally, a study conducted in Kenya also showed increased mental health symptoms among HCPs during the same period [12]. Despite these reported mental health concerns among HCPs during the pandemic, very few studies detail the lived experiences of frontline workers. Further, interventions of mental health support are majorly personalized approaches that require HCPs to initiate contact for mental health support. However, there are limited data on the effects of this approach [13]. An in-depth understanding of health providers’ lived experiences may give crucial evidence that could inform mental health intervention and document areas of improvement.

In Kenya, a multitasking force national emergency response committee comprising health, security, education, and transport coordinated the COVID-19 response by selecting public and private facilities, laboratories, and isolation centers, including guideline development for case management [14]. Further, the government adopted several strategies to respond to the pandemic, including a ban on international travel, a ban on social gatherings and meetings, and dawn-to-dusk curfews. In this regard, while the rest of the population reduced their exposure to infected individuals, health professionals worked in direct interaction with infected patients and were subject to a greater risk of infection themselves, with HCPs in some settings reporting high levels of anxiety, depression, insomnia, and burnout [12,15]. HCPs were on the front line of fighting the COVID-19 pandemic and were exposed to hazards, including pathogen exposure, long working hours, psychological distress, fatigue, occupational burnout, and stigma, that put them at risk of mental health challenges [16]. In addition, COVID-19 posed a risk in the healthcare system due to a stretched workforce and limited health infrastructure [17]. Given the substantial anticipated burden of psychological disorders in the context of the COVID-19 pandemic, it is essential that the mental health response is given priority.

There were considerable calls to put in measures and management to support HCPs’ mental well-being and enhance their resilience given the burden of anxiety and stress during the pandemic [14,15]. During the pandemic, several other countries incorporated psychological support, majorly through digital tools, tailored activities, and self-feedback training [18,19,20]. In our country, at the beginning of the pandemic, there was no formal response plan for COVID-19, coupled with implementation barriers due to poorly resourced mental health systems, poor policies, and a lack of innovation [9,10,21]. Further, the mental health policy in Kenya highlights the need for social inclusion, mental health assessment, and strengthening the referral systems [22]. However, little is known about the implementation of these systems among HCPs. Moreover, the HCPs in our country exhibited low literacy levels on mental health that may have impeded their willingness to access mental health services [23]. Within our context and other low-resource settings, there are scant data on how frontline HCPs coped with COVID-19 in their line of duty. An in-depth qualitative understanding of HCPs’ lived experiences gives nuanced experiences and contextual factors that are crucial to informing mental health interventions in Kenya and other resource-limited settings.

This phenomenological study [24] aimed to understand HCPs’ lived experiences during the COVID-19 pandemic. This evidence is useful for low- and middle-income countries where human resources for health are especially constrained, to develop and revise the existing guidelines to support HCPs’ mental well-being.

## 2. Methods

### 2.1. Study Design

We conducted a descriptive phenomenological study through the lens of subtle realism to understand frontline HCPs’ lived experiences at the Kenyatta National Hospital during the COVID-19 pandemic. This qualitative approach uniquely focuses on explicitly understanding individuals’ lived experiences within their setting [25].

### 2.2. Study Procedures and Participants

This article presents data collected between April 2021 and August 2021. We conducted in-depth interviews (IDIs) among HCPs at Kenyatta National Hospital. We used stratified purposive sampling to recruit clinical care providers (doctors, clinical officers, nurses). We determined the strata by first identifying the subgroups of either IDU or non-IDU units and cadres; this stratification helped to ensure good representation and views from different experiences based on their units and roles. The sampling included those working in the infectious disease unit (IDU) (n = 20) and other units outside the infectious disease unit (non-IDU) (n = 40). The non-IDU units did not admit known COVID-19 cases; only patients who tested positive and had severe symptoms were admitted to the IDU unit. These non-IDU departments included outpatient clinics, the accident and emergency department, pharmacies, wards, psychosocial units, etc. Essentially, the HCPs in both units were at risk of COVID-19 since one received patients with unknown COVID-19 results, and for the other, COVID-19 positivity was ascertained. The sample size in this study was determined according to saturation, where new interviews did not meaningfully add to the themes already represented in the collected data, as in the existing literature [26]. Study participants who met the inclusion criteria were contacted via phone by the study research assistant. Healthcare providers were eligible if they were working at Kenyatta National Hospital, either in the IDU or non-IDU unit, and were willing to provide written consent for study participation. The healthcare providers were invited to participate in the study by reading and signing an online consent form. Thereafter, an online survey was sent to those who consented to providing the demographic information. Participants who completed the online survey forms were contacted, and in-depth interviews were conducted on the phone or using the Zoom platform.

### 2.3. Study Setting

This study was conducted at Kenyatta National Hospital (https://knh.or.ke (accessed on 20 July 2023)), Kenya’s largest teaching and referral hospital situated in the capital city of Nairobi. The hospital admitted a significant proportion of COVID-19 cases, and by April 2021 (third wave), there were 2979 positive cases [27].

### 2.4. Participant Recruitment and Data Collection

The HCPs were recruited by a research team member, based at Kenyatta National hospital, who introduced the study. A study research assistant then contacted the providers by phone and sent an online link to the HCPs to complete a self-administered online survey form on socio-demographic information that included demographic data (age, sex, average income, and known contact with a COVID-19 patient). After the completion of the demographic forms, the HCPs were invited for in-depth interviews. All the HCPs who were contacted completed the demographic survey and agreed to participate in the in-depth interviews remotely. The qualitative interviews were conducted by bachelor-level experienced qualitative researchers using a semi-structured questionnaire to understand changes in stress levels and feelings of anxiety and depression, the mental health measures taken, and suggestions for improving mental health services. To further clarify anxiety and depression in this study, we asked the HCPs whether they had experienced extreme worry or extreme sadness. The interview guides were piloted and tested to understand the relevance and flow of the questions (Supplementary Materials). The interviews were conducted remotely via phone or using the Zoom platform, as per the participant’s preference, at their preferred date and time. The data were collected at a secure, centrally located field office where the participant and interviewer only were present. The interviews were voice-recorded using a digital recorder and uploaded onto a password-protected computer. The interviews were transcribed verbatim and translated where necessary. The transcripts were uploaded onto Google Drive to share them with the larger team for contextual discussions.

### 2.5. Rigor and Quality

To ensure both the quality of the evidence and the credibility of our findings [28,29], we assessed multiple perspectives from different cadres and departments to avoid solely relying on the view point of one group to increase the confirmability of our research findings.

### 2.6. Reflexivity

In this study, the data collection and analysis were conducted by two experienced social scientists with a non-clinical background. The participants’ awareness of the interviewers’ non-clinical background could have positively impacted their willingness to talk freely about their experiences.

### 2.7. Data Analysis

We used a combined method of content analytics and a thematic approach to analyze our qualitative data. The interview transcripts were first reviewed for their accuracy and completeness and later coded according to the study objectives, supported by the Dedoose software (version 8.3.35) (Sociocultural Research Consultants, LLC, Los Angeles, CA, USA), a web-based application for managing, analyzing, and presenting our qualitative data [30]. Two team members (V.O. and P.O.) first read through 20% of the transcripts independently, applied the initial codes from the initial list, and identified new codes based on the emerging themes not included in the “initial list”. The qualitative researchers discussed the discrepancies during the first stage of the coding process. After reaching an intercoder consistency of 0.80 on all the major themes, the two analysts (V.O. and P.O.) read through all the transcripts and coded the interviews using an agreed-upon codebook. The data were categorized into broader themes (mental health, impact of COVID-19 on social life, COVID-19 risk perception) and sub-themes (changes in stress levels, anxiety and depression, mental health support, and adequacy of mental health support) to begin to make sense of the healthcare providers’ lived experiences during the COVID-19 pandemic in the study design. In this article, we analyzed concepts of mental health experiences including experiences with stress, anxiety, depression, mental health support, and suggestions for improvement. As part of the analysis, direct quotations representative of the participants’ opinions were included. Our qualitative findings and reporting adhered to the COREQ guidelines [31].

## 3. Results

### 3.1. Demographic Characteristics of the Participants

The healthcare providers had a median age of 37 years (IQR 20–58), and 56% (34/60) of them were female. The majority of the participants were nurses, 18 (30%), and doctors, 11 (18%), respectively. The majority, 90% (54/60), of the respondents had known COVID-19 contact. The demographic characteristics of the respondents in our study are presented in Table 1.

### 3.2. Qualitative Findings

The HCPs reported changes in their stress levels, especially at the beginning of the pandemic, caused by the nature of their working environment and conditions, changes in their social life, and fears of COVID-19 infection. The respondents also described their experiences with high levels of anxiety and depression during the COVID-19 pandemic related to little knowledge of COVID-19, testing positive for COVID-19, exposure to risks, and the death of patients/colleagues. The HCPs also reported experiencing low or no changes in their stress levels due to the available mental health support from psychological counseling, debriefing sessions, and peer support, albeit concerns of privacy and confidentiality and ad hoc support varied across departments. The interviews took an average of 30–40 min. The themes and illustrative quotes are described in the following section.

### 3.3. Changes in Stress Levels Related to the Working Environment

The healthcare providers reported an increase in their stress levels due to working in a high-risk environment, associated with fear of COVID-19 infection, a high demand for patient care, and increased risk of contracting COVID-19. The HCPs reported that the departmental shifts (providers being moved to different departments within the IDU and non-IDU areas) and workload caused stress. The HCPs reported that adapting to changes, including wearing personal protective equipment, maintaining COVID-19 guidelines, and working within strict timelines, was a major challenge at the beginning of the pandemic.

“*Yeah, it is more stressful because there is a lot of pressure at work especially you get that big people [government officials] have their patients in the ward, they keep on pushing and making sure that they get services not within the timelines that are set in the guidelines they want to get their results within an hour or even 30 min… So sometimes there is so much stress because there is too much pressure from the ministry and from within the hospital. You feel like you want to resign from work. The motivation of working is not there.*”

[Female, laboratory technologist, IDU]

The changes brought about by COVID-19 in the hospital also limited patient care, especially among the non-IDU staff. The providers mentioned that it was their role to take care of patients, and whenever there were situations that compromised the quality of patient care, they felt affected. For instance, the introduction of mandatory pre-surgery COVID-19 tests before admission into theatre led to delays in procedures since not all patients could afford the tests, causing delayed surgeries. Additionally, because elective theater cases were not prioritized during the pandemic to manage overcrowding, some patients were restless, and not being able to support the patients at their time of need affected the HCPs’ mental health.

“*So, the mental anguish, I think, has been there. So, there has been an element from a patient perspective, delay in care… acute cases, denial of care delay in elective services to just overcrowding, limited staff, to the staffing in terms of the burnouts, being just extremely on the edge because of the COVID itself and then… it exposed the healthcare workers, to the mental anguish of being suspicious of having contracted the disease and finally having to confirm that you got the COVID-19. So, it’s been a roller coaster.*”

[Male, medical doctor, non-IDU]

Consequently, the HCPs added that being moved to other departments like the IDU caused fears and stigma, as the areas were perceived as “very high-risk” and contributed to increased stress levels. The HCPs added that being treated differently by their colleagues who had been close to them before the pandemic contributed to stress.

“*I had a feeling that I was being sent there to die since I didn’t have information and that fear and now you are sent to IDU where positive patients are and there was stigma around. We were working at night and we went to take tea there at night and when we left there, the colleagues boiled our cups. So, you see that kind of treatment just because someone is dealing with COVID, they want to treat differently. So, there was that stress, we were being stressed because of being treated differently.*”

[Male, laboratory technologist, IDU]

Further, the perception of being COVID-19-positive, which was associated with areas of work such as the IDU areas, also contributed to stress. The healthcare providers expressed that their colleagues perceived them as COVID-19-infected. This perception led to stigma because no one wanted to interact with them.

“*Another thing is that the other colleagues in the main hospital still view us as people who have COVID, when they see us go to the main hospital that stigmatization part of it is still here. So, it’s really taking a toll on us. There is an increased level of mental stress compared to pre-COVID.*”

[Male, nurse, IDU]

Importantly, some of the HCPs reported reduced stress levels and attributed it to the reduced workload brought about by COVID-19 measures that controlled overcrowding. Those who mentioned no change in stress levels described that they were coping with the new situation and felt they were not affected because of their coping mechanisms. Additionally, the providers who cited low stress levels reported high stress levels prior to the COVID-19 pandemic.

“*Before COVID my levels were like 8. Right now, my levels are like two, because there were so many patients. I checked all the patients, I checked all the files, work was a lot, work was tasking, but now life is good, how many patients do I have? I have 13 patients; I have 4 patients in the pediatric ICU and 9 patients in the burn unit. Honestly, my stress levels have reduced to two at most.*”

[Female, nutritionist, IDU]

### 3.4. Experiences of Depression and Anxiety

The HCPs reported experiencing anxiety and being extremely sad or extremely worried. Depression was reported by a few, perceived as a severe form of stress. Anxiety experiences were major at the beginning of the pandemic when there was little knowledge and guidance on COVID-19 management and there was the perception that “COVID-19 is death”. Existing misinformation about COVID-19 among providers and the community such as “COVID-19 causing reduced life span” also caused anxiety.

“*I was extremely worried about contracting COVID and the information out there was that when you contract COVID, you die. Some people saying ‘You will get sterilized’ and others were saying, your lifespan will reduce, if you recover, your lifespan will reduce, you see those misconceptions, it will reduce by ten years; so, I was worried.*”

[Male, laboratory technologist, IDU]

Anxiety was also characterized by fear of contracting the virus and fear of the unknown, e.g., “What happens when they get infected and what happens to their family and friends”.

“*Anxiety comes in terms of, what does tomorrow hold? What happens if I get COVID-19? What will happen to my children? What will happen to my spouse? What will my neighbor say if I tested positive and then my children are interacting with their children and then they will say it is you who is the source, the primary source of this COVID thing.*”

[Male, clinical officer, non-IDU]

The HCPs mentioned symptoms such as “lack of sleep”, “not feeling like talking to people”, “lack of appetite”, “eating a lot”, and “not feeling like going to work” while describing their experiences with anxiety and stress. As described by a medical doctor in the non-IDU area and a pharmaceutical technologist in the IDU area:

“*I know I have had problems sleeping, lately I have had a lot of insomnia. So, for the last year, I have been taking a lot of sleep medication just to sleep. I find myself so tired when I wake up. I used to be an 8 o’clock person to work. Nowadays I think I am at work at 9 and 9:30. I feel lethargic and tired, how do I say it… no morale very, very… low morale.*”

[Female, medical doctor, non-IDU]

“*You know there is a time when you don’t feel like talking to people, you don’t have an appetite… yeah those are the feelings that I have had in the past*”

[Male, pharmaceutical technologist, IDU]

Anxiety and depression symptoms were also experienced as a result of high exposure to COVID-19 risk, testing positive for COVID-19, and the inadequate supply of personal protective equipment (PPE) and hospital resources. For instance, the absence of oxygen in the IDU and the inadequate supply of PPE affected the HCPs’ mental health; as described by a respondent, “there were panic attacks”.

“*A lot there is so much demand for patient care there and also just anxious especially when we don’t have… sometimes we don’t have the right PPE and we still want patient care to be taken care of… So, we carry a lot of fatigue and anxiety when we are doing calls, especially with the crisis of not having PPEs sometimes or with the oxygen outrages*”

[Female, nurse, IDU]

### 3.5. Death of Patients and Colleagues as a Cause of Anxiety and Depression

Death caused anxiety and depression among the HCPs, who reported that it was traumatizing for them to watch patients and their colleagues die due to the COVID-19 virus. One respondent also mentioned that her colleague died by suicide, and that took a toll on her mental health.

“*Of course, I am not mentally stable, that is what I can say, because having mental health illness doesn’t have to be symptomatic but of course that worries me… we have lost close colleagues out of it so it has affected my usual stability because now I’m living in fear. If I lose my colleagues I go into panic. If I lose my seniors I go into a panic, so I have been quite unstable for this period that we have been having COVID.*”

[Female, clinical officer, IDU]

### 3.6. Changes in Social Life as a Mental Health Issue

Changes in social life brought about by the new COVID-19 restrictions such as social distancing and the cessation of movement presented major challenges. The HCPs reported that the inability to travel or meet family and friends affected their mental health due to a lack of social support. Face-to-face communication was reported to be key and telephone conversation insufficient. Other social interaction impediments occurred between the junior staff and the senior staff they resulted on for consultation, yet their interaction was limited. They reported that many of the senior HCPs worked remotely as per the country’s guidelines related to advanced age and preexisting illness. The social support system was perceived as “nearly zero” due to these restrictions.

“*Before we used to mix freely amongst ourselves, we used to share but now you find that if that social life is not there, you cannot sit with colleagues to give a story, to talk about your weekend experience and stuff like that… you may have something that you need to tell someone, not on phone but one on one so you are like now am going to sit with her and I don’t know where she was… let me just keep to myself and it is still eating you inside.*”

[Female, nurse, non-IDU]

Among other HCPs, financial constraints caused stress. The fact that the economy was shut down and cash flow was reduced was coupled with reduced extra sources of income and salary cuts for some providers. It was reported that the fact that several people lost their jobs during the pandemic and family and friends were dependent on the people who continued to work affected their mental health.

“*Being broke can give you hypertension if you try it. Depression and hypertension because as I told you we used to work in locum, so that was very stressful we have that financial aspect so despite the tax reduction that the government gave us, there is nothing much we can do in terms of effect. Secondly, now the risk that you have because of work, for work that you are not being paid for. So, to me, it was quite stressful.*”

[Female, medical doctor, non-IDU]

### 3.7. Experiences with Mental Health Support

Nearly half of the HCPs reported having either received mental health support from the hospital or been offered mental health services at the hospital. Those working in the infectious disease unit reported that psychological counselors were stationed at the IDU to support staff and patients. Other departments outside the IDU reported having department-specific mental health support, indicating that some departments incorporated psychological support while others did not.

The mental health support offered included face-to-face counseling, debriefing sessions among colleagues, peer counseling, toll-free numbers to contact when in need, and mental health webinars.

“*Okay in our section we have counselors with us, and when we are discussing especially on COVID experiences they help take care of stress, so if you feel you need them, they are readily available.*”

[Female, laboratory technologist, IDU]

Further, other HCPs reported having received mental health support from colleagues, family and friends, religious groups, and private counselors outside the hospital environment.

“*So, I am getting support from my colleagues and also my family members, they also pray for me and the church members, they always communicate to me… Yeah, those are the people who have given me the strength to move on.*”

[Female, nurse, non-IDU]

The HCPs reported not having received the mental health support described and not taking up the support due to their perceived “well-being”, mentioning that they were not at a point where they required the support. Another view expressed was a lack of knowledge about any mental health support available, which differed per department since some departments offered support while others did not.

“*In Kenyatta, they don’t have any support, my dear, what support do they have? They don’t have any support. Actually, the colleague that I told you about has been battling mental health for close to 15 years. The support offered was after making a plea to the chairman, a very nice gentleman. But for us, at Kenyatta, no one is interested in your mental health. In fact, as a resident, I have to pay consultation fees, and for everything else, I have to join the queue.*”

[Female, medical doctor, non-IDU]

### 3.8. Adequacy of the Mental Health Support

Almost all the HCPs reported the inadequacy of the mental health services provided. This inadequacy was due to the nature of the support offered. It was mentioned that the support offered was on an ad hoc basis and not an open forum for everyone. As they reported, people rarely want to talk about things that disturb them mentally, and therefore the “as-needed support” was not suitable for everyone. The providers also mentioned that mental health support was available at the beginning of the pandemic but over time there was laxity which caused inadequacies.

“*So, the patients, the psychological support has been resourceful but for the healthcare workers, I don’t know where it fits, I can’t say because if you ask me, ‘Do you need a counselor?’ I will tell you maybe I don’t need a counselor.*”

[Male, nutritionist, IDU]

The fact that the mental health support was not consistent across departments also caused inadequacies, as there was no standard provision of mental health services; for instance, the HCPs reported the availability of mental health support, while their colleagues reported a lack of support. The time spent per counseling session was reported to be low, as there were few counselors yet high demand for the counseling services. Other concerns reported by a few providers were that the colleagues offering mental health services were themselves “broken” and mentally overwhelmed and so could not fully maximize their potential while offering services.

“*Yeah but you see they are not really adequate because they are also overwhelmed. And you see for me to get to a point of reaching out to a counselor it means I’m reaching my limit of either anxiety or stress.*”

[Female, nurse, IDU]

“*Probably not, and especially departments or units where there is no psychological support, let me put myself in a department which doesn’t have the psychological support, the discussions that we normally have, so you could find that people could go into complete depression, others could not even be able to report to work. So, I would say the measures were not adequate. Yeah.*”

[Male, laboratory technologist, non-IDU]

The privacy and confidentiality of mental health services were also a concern. The HCPs felt they were not comfortable talking to their colleagues and preferred talking to providers outside the hospital environment. This challenge limited the uptake of mental health services in the hospital.

“*Offered I think yes, but taken up I think no. Yeah, if they have you can always go but ah! [sigh] I always do not go, let me handle myself in my way… how do you go to people who know you? Okay as much as they are supporting us, we are still colleagues… I cannot come to you and then meet you in the corridor the following day, I will be uncomfortable. So, as much as it has been offered, taking it up is just a challenge.*”

[Female, social worker, non-IDU]

A health provider clarified that mental health challenges arose due to the perception of one’s environment, and therefore it was important to take care of one’s social needs, which would translate to mental well-being, stating that:

“*Mental health comes from our perception of the environment we live in. So, let us address the basics before we go to mental health. There are scientific theoretical models for example the social determinants of mental health, so if you take care of someone’s physiological needs, like security, food, to have good medical care those are things that will take care of the mental health status. Let us address the basics, and then you will end up addressing the mental health conditions like anxiety, depression, fears, and unhealthy behaviors of health care providers.*”

[Male, nurse, non-IDU]

### 3.9. Recommendations for Mental Health Services

The HCPs reported the need to improve mental health services not only specifically due to the COVID-19 pandemic period but for their general well-being, given the nature of their job. The following recommendations were made to improve mental health support: sensitization of the staff on the importance of mental well-being by conducting mental health outreach, the utilization of proactive mental health screening tools for all providers to promptly address symptoms and not relying on a need basis, the need to partner with counseling facilities outside the hospital environment to enhance privacy and confidentiality, the development of a mental health support team at each unit in the hospital, the need to learn better ways of approaching people on mental health issues due to the “perceived stigma on mental health”, and designing innovative strategies to improve mental-healthcare-seeking behaviors. A health provider working in the non-IDU area highlighted the need to have a counseling unit in the hospital.

“*Okay within our set up even with or without COVID, there should be a way of debriefing our issues, we should have a psychotherapy kind of a thing, a counseling in our unit, not necessarily doors but they could be coming in weekly, for some kind of group therapy… okay some conditions are very traumatizing, like you may get a very helpless situation where you can’t help the patient and with that, you carry it in your heart for several days even years, it increases your fear in life, you put it like “what if it was me?”, We may have a debriefing once in a while but we don’t have such kinds of initiatives in our department. We don’t.*”

[Female, clinical officer, non-IDU]

## 4. Discussion

This study used qualitative analysis to explicate the mental health experiences of HCPs during the COVID-19 pandemic at the Kenyatta National Hospital, a key national COVID-19 management center. The HCPs reported experiences with stress and anxiety, especially at the beginning of the pandemic, as well as depression. The healthcare providers reported the demand for patient care in a high-risk environment coupled with very limited knowledge and guidance on the management of COVID-19 as reasons for anxiety. Additionally, the healthcare providers reported fear of COVID-19 infection and uncertainty about the outcome of infection [32]. The changes brought about by COVID-19 measures such as the cessation of movement due to curfews and physical distancing; attendant economic hardships; and the death of their colleagues contributed to stress. The mental health support provided was reported to be inadequate, as it was administered on a need basis, and there were concerns about breaches of privacy and confidentiality, provoking fear of stigmatization.

The HCPs reported stress and anxiety due to changes in their working environment. In 2020, HCPs globally exhibited high levels of anxiety and depression [33,34,35]. Additionally, the WHO reported a 25% increase in the prevalence of anxiety and depression due to COVID-19 [36]. Several studies have also shown changes in levels of stress, anxiety, and depressive symptoms during the pandemic [37,38]. However, very few studies describe these concerns qualitatively. In our study, the HCPs mentioned misinformation such as COVID-19 causing a “reduced life span” or “being sterile” as some existing misinformation that affected their mental health. In addition, the high demand for patient care, working in a high-risk environment, and caring for COVID-19 patients affected the HCPs’ mental health. On the contrary, a study in China found low stress levels among HCPs caring for COVID-19 patients, portraying professional devotion and altruism [39], which were different in our setting. In addition, the COVID-19 pandemic put additional demands on an already stretched healthcare system in Kenya, with reports on shortages of personal protective equipment and testing kits, coupled with strained human resources [40], which also contributed to the increased stress levels among the HCPs in our setting.

Changes in social life and the death of colleagues and patients were significant mental health issues. The WHO reported changes with the COVID-19 outbreak such as physical distancing and the cessation of movement that impacted people’s social interactions [41]. The HCPs mentioned that they relied on their seniors for guidance and consultation, but due to COVID-19 restrictions, they were unable to receive support. Many senior HCPs worked remotely as per the country’s guidelines related to advanced age and preexisting illness. In addition, interactions with friends and families were limited, yet some relied on them for psychological support. Similarly, other studies have also shown that interpersonal, intrapersonal, and organizational factors of COVID-19 have an impact on HCPs’ mental well-being [42]. The HCPs also reported that the high levels of morbidity and mortality among colleagues and patients affected their mental health. In addition, COVID-19-related deaths have been shown to contribute to mental health disorders given the mitigation measures limited grief within social networks [43]. In addition, a study conducted in the US highlighted the need for HCPs to put in individual preparation for deaths for their health grieving and the need for the health system to support workers in their anticipatory and realized grief [44]. In our study, none of the HCPs reported grief management, hence the high number of deaths causing panic and stress, as the HCPs mentioned “feeling they were next”. Therefore, there is an urgent need for personal and professional grief management strategies in the healthcare system to support healthcare providers’ mental health.

Experiences with mental health support and recommendations were also key measures for dealing with the COVID-19 pandemic. In our study, the HCPs reported psychological support through debriefing sessions, counselors, peer support, and toll-free numbers. These strategies supported the health workers in handling their anxiety and stress levels. Comparably, a systematic review showed evidence of these interventions during the pandemic outbreak that was in agreement with our findings [45]. Consequently, some of the providers mentioned reduced stress and anxiety levels due to the reduced workload brought about by the pandemic and the support strategies that were put in place. However, the providers reported concerns about and challenges with the existing support mechanisms, such as support being on an “ad hoc basis” and privacy and confidentiality concerns. In this context of COVID-19, a study in Indonesia illustrated coping mechanisms among health workers that reduced stress levels such as having a positive attitude, adequate knowledge, and family support [46]. In our setting, the HCPs had challenges with misinformation and inadequate social support, which informed mental health issues. In addition, the lessons learned in Kenya during HIV and Ebola pandemics could be key in developing strategies to support HCPs’ mental health, such as the availability of counselors, managed risk “allowances” and compensation, and dispelling misinformation [47]. Further, the HCPs gave important recommendations that could improve mental health in their context while dealing with pandemic outbreaks. These recommendations underscore participatory interventions that could support mental well-being among providers.

To the best of our knowledge, this study is unique by not only describing HCPs’ mental health experiences but also exploring the nature of mental health support available and suggestions for improvement. This information may be used to revise the existing guidelines to adequately support HCPs. We found critical in-depth experiences that described increased mental health challenges during the pandemic. Importantly, some providers had reduced stress levels during the pandemic, which not only associates these experiences with the pandemic but also with the nature of clinical care. In addition, our study reveals that an inability to offer patients high-quality care due to challenges such as inadequate hospital resources, e.g., oxygen, impacted the providers’ mental health. Similarly, other studies have shown increased mental health disorders and highlight the need to increase resource allocation to ensure the delivery of high-quality service [48,49]. Our study also highlights gaps in grief management following the death of patients and colleagues that call for mechanisms for coping with bereavement within healthcare systems. In addition, we also found privacy and confidentiality as challenges within the existing mental health support offered. Interventions seeking to improve mental health services among HCPs may explore innovative strategies that are private, can be adopted consistently, and do not put HCPs at risk of stigma.

This study had an important strength. Principally, the use of qualitative approaches offered a detailed picture in understanding the lived experiences of mental health among HCPs working directly or indirectly with COVID-19 patients during the pandemic and ultimately how their stress and anxiety levels changed. The primary limitation of this study was that we did not incorporate mental health assessment tools; therefore, the nature of stress, anxiety, and depression described was based on the experiences reported by the HCPs. In addition, there exists potential for social desirability bias, and given the nature of this qualitative study, our results are not generalizable; rather, we provide insights into the HCPs’ lived experiences during the COVID-19 pandemic. Lastly, these findings only describe the experiences within the Kenyan healthcare system context, which may differ from the experiences outside our health system environment.

## 5. Conclusions

In summary, the qualitative findings in working with the HCPs demonstrated their critical experiences of the COVID-19 pandemic and essential suggestions for improving mental health services. The HCPs reported mental health issues related to the work environment, social life, stigma, and the death of patients and colleagues, and their mental health support was largely reported as inadequate. Framed within the context of the pandemic, health professionals had to deal with high stress levels and anxiety, stigma, and changes in their working environment. Interventions to improve mental health response in our resource-constrained setting require institutional practices that address context-specific challenges, such as the time spent during counseling sessions, privacy and confidentiality, and the consistent provision of mental health support and grief management. Future studies may use mental health assessment tools to evaluate post-traumatic stress disorder, anxiety, burnout, and depression to explore the magnitude and variety of mental health outcomes. In addition, mental health program studies may explore the effectiveness of interventions designed to support HCPs’ mental health, as well as experiences among divergent health system contexts.

## Figures and Tables

**Table 1:** Participant demographic characteristics.

Demographic Characteristics	Clinical Officer (N = 3)	Medical Doctor (N = 11)	Lab Technologist (N = 7)	Nurse (N = 18)	Pharmaceutical Technologist (N = 5)	Other ^1^ (N = 16)	Total (N = 60)
**Age**							
Mean (SD)	38.0 (2.65)	35.5 (8.74)	38.7 (6.10)	41.4 (11.1)	40.0 (6.36)	35.1 (7.97)	38.1 (8.88)
Median [Min, Max]	39.0 [35.0, 40.0]	35.0 [27.0, 58.0]	38.0 [32.0, 48.0]	45.0 [20.0, 58.0]	38.0 [32.0, 47.0]	32.0 [26.0, 53.0]	37.0 [20.0, 58.0]
**Sex**							
Female	2 (66.7%)	8 (72.7%)	4 (57.1%)	12 (66.7%)	2 (40.0%)	6 (37.5%)	34 (56.7%)
Male	1 (33.3%)	3 (27.3%)	3 (42.9%)	6 (33.3%)	3 (60.0%)	10 (62.5%)	26 (43.3%)
**Income**							
≤50,000	0 (0%)	0 (0%)	0 (0%)	2 (11.1%)	0 (0%)	4 (25.0%)	6 (10.0%)
50,000–200,000	3 (100%)	5 (45.5%)	7 (100%)	14 (77.8%)	5 (100%)	12 (75.0%)	46 (76.7%)
≥200,000	0 (0%)	6 (54.5%)	0 (0%)	2 (11.1%)	0 (0%)	0 (0%)	8 (13.3%)
**Contact with COVID-19-positive patient**							
Yes	3 (100%)	10 (90.9%)	6 (85.7%)	17 (94.4%)	5 (100%)	13 (81.3%)	54 (90.0%)
No	0 (0%)	1 (9.1%)	1 (14.3%)	1 (5.6%)	0 (0%)	3 (18.7%)	6 (10.0%)

^1^ Health records officer, clinical psychologist, social worker, nutritionist.

## Data Availability

The individual participant data that underlie the results reported in this article, after deidentification, are available following publication and under the appropriate data sharing agreements. The data are available to researchers who provide a methodologically sound proposal.

## Supplementary Materials

The following supporting information can be downloaded at: https://www.mdpi.com/article/10.3390/bs14050351/s1, In-depth interview guide for health care providers.
