# Supplementary material for: The Effects of the COVID-19 Pandemic on Healthcare Providers’ Mental Health: Experiences at Kenyatta National Hospital, Kenya
Source: Behav Sci (Basel). 2024 Apr 23;14(5):351. doi: 10.3390/bs14050351 (PMC11118936; doi:10.3390/bs14050351)
Supplement: Supplementary file 1 [file behavsci-14-00351-s001.zip › behavsci-2897836-supplementary.pdf]

Supplementary Materials: In-depth interview guide for health care providers

Frontline Health Care Providers' Experiences with COVID-19 at Kenyatta National Hospital, Kenya – A Phenomenological Study

Introduction: Welcome to our interview today, we are grateful that you have volunteered to participate. We are conducting some interviews with health care providers who work with patients during this time of the COVID-19 pandemic. We are going to talk with 60 providers like yourself. The information we get from you will inform the development of an intervention to support healthcare providers respond better to the COVID-19 pandemic. Please feel free to express your views during this interview. Before we start, do you have any questions?

I. <u>Ice Breaker</u>

1. To begin, please tell me about your role in this hospital.

   Probes:
   - What do you do on a day-to-day basis?
   - Where do you work within the hospital?
2. How long have you been in this role? (years/months)
3. How has your social life changed by COVID-19?
4. How has your work been affected by the COVID epidemic?

   Probes:
   - Changes in numbers of patients (workload)?
   - Changes in type of patients?
   - Clinic adaptations to deal with COVID-19?

II. <u>COVID-19 Knowledge</u>

How would you describe your knowledge about COVID-19? How does this affect how you manage your patients? What fears do you have regarding COVID-19?

III. <u>Risk perception</u>

5. Based on your current duties do you feel at risk of COVID-19? Why?

Probes:
- How does your risk compare with that of other health care providers?
- Where do you work within the hospital?
- Do you suspect you or other health care providers could be infected with COVID-19?

IV. <u>COVID-19</u>

6. How are you protecting yourself from COVID-19?

Probes:
- Differences in measures taken at work and home?
- In your opinion do you feel that you have enough personal protective equipment? Masks?

- Do you feel that these measures taken are adequate?

7. How do you protect others from COVID-19? Probes: Patients? Family members?

V. Mental Health

8. Compared to before COVID-19, how would you describe changes in your stress levels? why

Probes:
- Have you had a feeling of Anxiety or depression? (have you experienced a feeling of extreme worry or extreme sadness)
- How is the family (where applicable) coping with the changes/ how is it affecting them?
- What kind of support have you been offered regarding your mental health?
- Do you feel that this support and measures taken are adequate?

VI. Stigma

9. How is your family and community currently treating you? How is it different from how they were treating you before COVID-19?

VII. Suggestions for Improvement

10. What suggestions do you have that can be done to support you and other health care providers better during this COVID-19 pandemic?

VIII. Conclusion

11.We have reached the end of our interview. Do you have anything else to add?

[Interviewer]: *Thank you again for participating in our study!*
